# Announcing the launch of Protein Data Bank China as an Associate Member of the Worldwide Protein Data Bank Partnership

**DOI:** 10.1107/S2059798323006381

**Published:** 2023-08-10

**Authors:** Wenqing Xu, Sameer Velankar, Ardan Patwardhan, Jeffrey C. Hoch, Stephen K. Burley, Genji Kurisu

**Affiliations:** a Protein Data Bank China, ShanghaiTech University and National Facility for Protein Science in Shanghai, Shanghai, People’s Republic of China; b Protein Data Bank in Europe, European Molecular Biology Laboratory, European Bioinformatics Institute, Wellcome Genome Campus, Hinxton, Cambridge CB10 1SD, United Kingdom; c Electron Microscopy Data Bank, European Molecular Biology Laboratory, European Bioinformatics Institute, Wellcome Genome Campus, Hinxton, Cambridge CB10 1SD, United Kingdom; d Biological Magnetic Resonance Data Bank, UConn Health, Farmington, CT 06030-3305, USA; e Research Collaboratory for Structural Bioinformatics Protein Data Bank, Institute for Quantitative Biomedicine and Department of Chemistry and Chemical Biology, Rutgers, The State University of New Jersey, Piscataway, NJ 08854, USA; f Research Collaboratory for Structural Biology Protein Data Bank, San Diego Supercomputer Center, University of California San Diego, La Jolla, CA 92093, USA; g Protein Data Bank Japan, Institute for Protein Research, Osaka University, Osaka 565-0871, Japan; h Protein Data Bank Japan, Protein Research Foundation, Minoh, Osaka 562-8686, Japan; University of Cambridge, United Kingdom

**Keywords:** macromolecular crystallography, nuclear magnetic resonance, three-dimensional cryo-electron microscopy, Protein Data Bank, Biological Magnetic Resonance Bank, Electron Microscopy Data Bank, Worldwide Protein Data Bank

## Abstract

The Protein Data Bank (PDB) is the single global archive of atomic-level, three-dimensional structures of biological macromolecules experimentally determined by macromolecular crystallography, nuclear magnetic resonance spectroscopy or three-dimensional cryo-electron microscopy. The PDB is growing continuously, with a recent rapid increase in new structure depositions from Asia. In 2022, the Worldwide Protein Data Bank (wwPDB; https://www.wwpdb.org/) partners welcomed Protein Data Bank China (PDBc; https://www.pdbc.org.cn) to the organization as an Associate Member. PDBc is based in the National Facility for Protein Science in Shanghai which is associated with the Shanghai Advanced Research Institute of Chinese Academy of Sciences, the Shanghai Institute for Advanced Immunochemical Studies and the iHuman Institute of ShanghaiTech University. This letter describes the history of the wwPDB, recently established mechanisms for adding new wwPDB data centers and the processes developed to bring PDBc into the partnership.

## Abstract 2

The formal launch of Protein Data Bank China (PDBc) is announced, and the history of the wwPDB, recently established mechanisms for adding new wwPDB data centers and the processes developed to bring PDBc into the partnership are described.

In 1971, the Protein Data Bank (PDB) was established as a global public good jointly by Brookhaven National Laboratory (BNL) in the United States and Cambridge Crystallo­graphic Data Center in the United Kingdom (Protein Data Bank, 1971 ▸). It was managed by BNL until 1998, with continuous support from US federal funders. Between 1999 and 2003, the PDB was managed by the Research Collaboratory for Structural Bioinformatics Protein Data Bank (RCSB PDB; https://www.rcsb.org), headquartered at Rutgers University (Berman *et al.*, 2000 ▸), again with continuous support from US federal funders. Between 1999 and 2003, Protein Data Bank in Europe (PDBe; https://www.pdbe.org) and Protein Data Bank Japan (PDBj; https://www.pdbj.org) coordinated deposition and biocuration efforts with RCSB PDB.

Since 2003, the PDB archive has been managed by the wwPDB (Berman *et al.*, 2003 ▸), an international nongovernmental organization founded by three regionally funded wwPDB data centers: RCSB PDB (United States), PDBe (United Kingdom and Europe) and PDBj (Japan). Two method-focused wwPDB data centers, the Biological Magnetic Resonance Bank (BMRB; https://bmrb.io) and the Electron Microscopy Data Bank (EMDB; https://www.ebi.ac.uk/emdb), joined the wwPDB in 2006 and 2021, respectively (wwPDB Consortium, 2019 ▸). wwPDB partners adhere to the FAIR principles of Findability, Accessibility, Interoperability and Reusability (Wilkinson *et al.*, 2016 ▸), and ensure that all archival data can be accessed at no charge and with no limitations on usage under the most permissive Creative Commons CC0 1.0 Universal License (https://creativecommons.org/publicdomain/zero/1.0/). At present, wwPDB members jointly manage three Core Archives overseen by wwPDB-designated Archive Keepers: the PDB (Archive Keeper: RCSB PDB), EMDB (Archive Keeper: EMDB) and BMRB (Archive Keeper: BMRB). wwPDB operations are guided by an international advisory committee (wwPDB AC; https://www.wwpdb.org/about/advisory) consisting of an independent chair, representatives appointed by each wwPDB data center and experts drawn from the macromolecular crystallography (MX), three-dimensional cryo-electron microscopy (3DEM) and nuclear magnetic resonance (NMR) spectroscopy communities. The wwPDB AC meets annually to review the health and wellbeing of the three Core Archives. Annual meeting presentations and reports are publicly available online.

With great foresight, PDB data were made freely available with no limitations on usage to the global scientific community (including for-profit users) at the time of launch in 1971. This pioneering online digital data resource, the first of its kind in biology, demonstrated the importance of free dissemination of scientific information to support both basic and applied researchers. A working group formed by the International Union of Crystallography (IUCr) defined the guidelines for 3D biostructure data deposition (Commission on Biological Macromolecules, 1989 ▸). These guidelines formed the basis for the deposition of atomic coordinates and experimental data in the PDB. Today, most government and private bioscience funders and scientific journals mandate the deposition of 3D biostructure data as a condition for either funding or publication. The central role played by the PDB within the worldwide biodata ecosystem was recently recognized by its designation as a Global Core Biodata Resource ‘of fundamental importance to the wider biological and life sciences community and the long-term preservation of biological data’ by the Global Biodata Coalition (https://globalbiodata.org).

When the wwPDB was launched 20 years ago, significant growth in the PDB archive was eagerly anticipated. At that time, each wwPDB data center accepted 3D biostructure depositions through independently operated data deposition/validation/biocuration (data-in) systems. To accommodate the increased demand for data archiving, wwPDB members initiated the development of a single global software system for data-in and started planning for the extension of the franchise to new PDB data centers for the management of depositions originating from emerging economies. Both steps were strongly endorsed by the wwPDB AC.

In 2015, the wwPDB launched *OneDep* (Young *et al.*, 2017 ▸) as a single global system for complete deposition, rigorous validation (Gore *et al.*, 2017 ▸; Feng *et al.*, 2021 ▸) and expert biocuration of 3D biostructures (Young *et al.*, 2018 ▸) in the PDB, EMDB and BMRB wwPDB Core Archives. The *OneDep* software system also supports the archive-wide remediation of existing structures (see, for example, Shao *et al.*, 2021 ▸) and the secure transfer of information among wwPDB data centers. Since it was launched, *OneDep* has been continuously maintained and upgraded by a wwPDB software developer team to ensure the robust capture of data from new and emerging structure-determination methods. At present, the wwPDB is working with major structural biology software providers to develop more automated and efficient deposition procedures and to capture data from standardized workflows commonly used within the MX and 3DEM communities.

Each wwPDB data center manages its own instance of *OneDep*. Initially, incoming structures were assigned to wwPDB data centers on a regional basis as follows: RCSB PDB, America and Oceania; PDBe/EMDB, Europe and Africa; PDBj, Asia and the Middle East. During 2017, RCSB PDB, PDBe/EMDB and PDBj processed 6206, 4044 and 2799 new depositions, respectively (a total of 13 049; MX, 11 915; 3DEM, 674; NMR, 460). In 2022, RCSB PDB, PDBe/EMDB and PDBj processed 7020, 4811 and 4214 new depositions, respectively (a total of 16 344; MX, 10 650; 3DEM, 5407; NMR, 287). Annual depositions of 3DEM experimental maps to EMDB were as follows: 1448 in 2017 (including 674 maps associated with PDB depositions and 774 maps without atomic coordinates) and 8519 in 2022 (including 5407 maps associated with PDB depositions and 3112 maps without atomic coordinates). Annual experimental data depositions to BMRB for NMR macromolecular structures were 460 in 2017 and 287 in 2022. The growth in the number of PDB depositions originating from Asia is primarily the result of increased investment in research and development within the People’s Republic of China (PRC). In 2022, structural biologists working in the PRC contributed 3118 new PDB structures (accounting for ∼65% of all depositions originating from Asia). Reflecting the impact of the resolution revolution, 3DEM structure depositions to the PDB originating from the PRC in 2022 numbered 1538 (versus 1539 MX structure depositions and 41 NMR structure depositions originating from the PRC in 2022).

wwPDB operations are governed by an international agreement (https://www.wwpdb.org/about/agreement) that was most recently renewed at the beginning of 2021. The new ‘Charter of the wwPDB’ defined all five existing members as Core Members of the wwPDB and established new processes for admitting new Associate Members and advancing them to Core Membership. These procedural changes were reviewed and endorsed by the wwPDB AC. Effective 1 February 2022, PDBc was admitted to the wwPDB partnership as its first Associate Member. During the remainder of 2022, to support the launch of PDBc, remote training, encompassing orientation and policy matters, was provided by RCSB PDB, PDBe and EMDB. Thereafter, PDBj provided full-time, hands-on training on-site for two PDBc biocurators in Osaka. RCSB PDB, PDBe and EMDB helped to mitigate the impact on PDBj by processing overflow depositions from Asia during PDBc on-site training.

Following two months of intensive training at PDBj, the PDBc biocurators returned to the National Facility for Protein Science in Shanghai and began processing PDB depositions originating from within the PRC using a new PDBc *OneDep* instance (currently managed by PDBj). As of the end of 2022, the PDBc biocurators had processed 297 new PDB depositions, representing ∼10% of the 3D biostructure depositions from the PRC for the year (∼7% of all Asian depositions). During 2023, it is expected that PDBc will process most, possibly all, depositions to all three wwPDB Core Archives made by structural biologists working in the PRC.

The wwPDB is excited to formally announce the launch of PDBc, coinciding with the 20th anniversary of continuous wwPDB operations serving more than 50 000 structural biolo­gists around the world and many millions of data consumers based in nearly every sovereign country recognized by the United Nations.
